# Using Written Narratives in Public Health Practice: A Creative Writing Perspective

**DOI:** 10.5888/pcd11.130402

**Published:** 2014-06-05

**Authors:** Tess Thompson, Matthew W. Kreuter

**Affiliations:** Author Affiliation: Matthew W. Kreuter, Washington University in St Louis, Health Communication Research Laboratory, St Louis, Missouri.

## Abstract

Narratives have become an increasingly common health communication tool in recent years. Vivid, engaging writing can help audiences identify with storytellers and understand health messages, but few public health practitioners are trained to create such stories. A transdisciplinary perspective, informed by both creative writing advice and evidence-based public health practices, can help public health professionals use stories more effectively in their work. This article provides techniques for creating written narratives that communicate health information for chronic disease prevention. We guide public health professionals through the process of soliciting, writing, and revising such stories, and we discuss challenges and potential solutions.

People attend, remember, and are transformed by stories.

— Mary Pipher, *Writing to Change the World* (1)

## Background

Narratives have become an increasingly common health communication tool, but few public health practitioners are trained to create them (2). The writing style suitable for technical reports, program summaries, and grant proposals is very different from the simple, vivid writing that helps audiences connect with storytellers and understand health messages. We believe that a transdisciplinary perspective that creates new approaches to problem-solving can give public health professionals insights from creative writing and narrative communication that will help them use stories effectively (3). This article draws from one author’s experience teaching creative writing and from the other author’s expertise in narrative health communication to provide techniques for creating written narratives that communicate health information for chronic disease prevention.

Narratives are a natural, engaging means of communication. For public health purposes, a narrative is “any cohesive and coherent story with an identifiable beginning, middle, and end that provides information about scene, characters, and conflict; raises unanswered questions or unresolved conflict; and provides resolution” (4). Public health interventions may use fictional stories, authentic stories, or composite stories, which are realistic stories based on multiple narratives (5). Narrative interventions in various media have been used to promote health behaviors such as controlling hypertension (6), quitting smoking (7,8), receiving mammograms (9), and preventing skin cancer (10). One study, for example, found that reading narrative information about skin cancer increased the chance of engaging in preventive behaviors compared with reading an informational text (10). In another study, tailored success stories delivered through the Internet effectively promoted smoking cessation (8). Narratives may hold potential for targeting hard-to-reach populations and reducing health disparities (11). Narratives can also be used to disseminate information about chronic disease to policy makers (12) and to share stories about effective programs (2,13).

One strength of narratives is their ability to engage audiences. Writer and psychologist Mary Pipher contends, “Stories are particularly effective in places where logical statements would inspire argument. If a story is well conceived and well told, listeners are likely to experience emotions that soften their positions and enable them to consider the speaker’s point of view” (1). Research has found that, compared with predominantly informational methods of presentation, narratives can lead to stronger emotional reactions, greater identification with the person sharing the messages, and increased engagement (14).

These effects are consistent with theories of narrative and health behavior. Green and Brock’s Transportation-Imagery Model contends that stories are effective because they “transport” the audience through use of vivid imagery and identification with characters (15). Social Cognitive Theory (16) holds that behavioral modeling, including firsthand experiential narratives, can provide an audience with role models who demonstrate a new behavior. Zillmann’s Exemplification Theory holds that using exemplars — or characters whose stories serve as concrete, vivid examples — can increase emotional engagement with health-related stories and lead to enhanced memory of messages over time (17). Busselle and Bilandzic examined dimensions of audience engagement, including narrative understanding, attentional focus, emotional engagement, and narrative presence, and they concluded that engagement and enjoyment affect story-related beliefs and attitudes (18). Moyer-Gusé’s Entertainment Overcoming Resistance Model asserts that involvement with story characters may lead people to be less resistant to messages (19).

Written narratives in particular, whether shared in printed materials or over the Internet, can be used to communicate health information to a diverse audience. One objective of *Healthy People 2020* is to employ health communication strategies for improving population health outcomes (20). *The Guide to Community Preventive Services* identified a range of evidence-based communication strategies, including small media, client reminders, educational materials, and social marketing (21). Although none of these strategies specifies the use of stories, stories can be embedded in or delivered through them.

### Shaping stories

Successful use of narrative to communicate health information requires a transdisciplinary approach that incorporates a mix of practical tools and techniques from creative writing and public health practice. In the spirit of Fix et al (22), who used insights from theater arts to enhance storytelling for patient education, we draw insights from creative writing to help public health practitioners use written narratives to influence health behavior.

These tools and techniques are designed for public health professionals working independently, but they may be even more effective when used in partnership with professional writers or editors. When funding is limited, English departments at colleges and universities may be able to suggest talented students who would like to volunteer or work on a limited budget to gain experience.

## Using Stories

### Deciding to use stories

Public health practitioners must first determine whether written narratives are an appropriate communication strategy, given their goals and intended audience. Stories in general may be appropriate for helping an audience overcome resistance to health messages or reluctance to change health-related behaviors. Stories may help readers process complex or highly emotional material (eg, information about cancer treatment) or overcome high levels of medical mistrust (23). Written stories in particular may be appropriate when using small media and other evidence-based methods for promoting health (21). For some audiences, however, such as audiences with low literacy levels or highly motivated audiences that prefer statistical information, formats other than written stories may be more appropriate.

After deciding to use stories, consider their purpose, the intended audience, and the type of stories to use. Stories can, for example, lead into an informational article, provide supplemental information in a brochure, or stand alone on a Web page. Having a clear purpose for a story will guide other aspects of the story, such as length and amount of detail.

Defining the audience is important because stories often derive their power from audience members’ identification with characters (14). It is often helpful if key characters are similar to the audience in a way relevant to the story’s purpose (eg, characters are age-eligible for breast cancer screening, if mammography is the goal).

Writers must also decide whether to use authentic stories, fictional stories, or composite stories. Authentic stories may have the benefit of being perceived as realistic, especially if they are labeled “real stories,” but research indicates that using fictional instead of authentic stories may not affect the story’s persuasiveness (15,24). Stories that are not written by professional writers will almost certainly need extensive editing (2). Creating fictional stories based on observations and experiences requires writers to 1) know their subject matter and audience well so they can provide realistic details and 2) use a voice that will be easily understood by the intended readers. Composite stories created from narratives gathered through formative research provide an opportunity to use realistic details that can be fictionalized and shaped into unified, factually accurate stories.

### Collecting stories

Authentic or composite stories should be collected systematically. Although there is no universally accepted protocol for developing health-related narratives, researchers have documented various formative processes that many public health practitioners find helpful. Wengraf provides a 3-step interview process to elicit narratives: first, ask the participant a general question about a topic (eg, “Tell me the story of your breast cancer”); next, ask them to elaborate on parts of their story (eg, “How did you tell your children about your diagnosis?”); and finally, probe with additional questions about topics of interest that did not arise naturally (25). Horner et al describe their process of using qualitative analysis of semistructured interviews with African American teenagers to learn about culturally sensitive narratives about HIV prevention behaviors (26). Fix et al provide a storytelling protocol that uses insights from theater arts to find appropriate storytellers, elicit compelling stories, and select the most effective stories for use in patient education (22).

Two protocols developed for generating print-specific narratives are especially helpful to public health practitioners. To share success stories about a cardiovascular screening program, Lewis et al developed a 10-step process to collect information used to create written narratives (13). The steps include specifying audience and purpose, soliciting story ideas from staff members, creating a standardized data collection form, conducting follow-up interviews to obtain additional details, and revising stories. The process described by Zwald et al (2) includes similar steps and extends those ideas by providing examples of tools used for story development, including a Web-based form with questions to collect specific details about programs and a follow-up interview script to solicit additional information.

It is important to use authentic and composite stories ethically (23). When collecting authentic stories, obtain consent from storytellers and tell them exactly how their stories will be used. Obtain explicit written permission to use identifiable images or personal health information. Storytellers should be told whether their stories will be used in their entirety or combined to form composite narratives. If using fictional stories, be sure to create stories that are nonstigmatizing and culturally appropriate; it is important to be sensitive to the difference between using cultural information to inform stories and using stereotypes that may be offensive or overly simplistic (5). It is also important to avoid using stories — however compelling — that include inaccurate or misleading information (23).

### Creating stories

Once formative research has been conducted, techniques from writing manuals and creative writing workshops can help public health professionals use the information to create vivid stories that connect with readers. The following principles of good storytelling provide a starting point for public health professionals who would like to incorporate stories in their work. Although many recommendations come from books and workshops about writing fiction, they also apply to composite and authentic narratives. (See Appendix for a sample story.)

**Choose a point of view.** In *Writing Fiction*, Burroway says that the concept of point of view can be summed up by the following questions: “Who speaks? To whom? In what form? At what distance from the action?” (27). Most health narratives use either a first-person perspective (“I’ll never forget my first mammogram”) or third-person perspective (“Rita Evans will never forget her first mammogram”). First-person narration can lend intimacy and promote engagement (28), but third-person can be used to weave together multiple stories or multiple people’s points of view (29). It can be helpful to try both first-person and third-person point of view and see which is most effective for a particular story or intended audience (29). Consider also “distance from the action”: are cancer patients talking about current treatment or diagnoses that happened years ago? Will the story focus on experiences of a patient, or is the narrator a parent or family member watching someone else face a health issue? It is often best to align the point of view in the story with that of the target audience; for example, a story designed to provide information to partners of cancer patients is probably best told from the perspective of a patient’s partner.

**Establish the conflict.** Conflict is “the fundamental element in fiction” (27). In fictional narratives, conflict is a crucial part of plot: characters must face their conflicts and reach a resolution. Not all health narratives have the complex and dramatic conflicts of a bestselling novel or a blockbuster movie, but the most effective stories will involve a character facing a conflict that is eventually resolved. A conflict may be internal (eg, dealing with the fear of getting a mammogram) or external (eg, dealing with an uncommunicative doctor). The stakes in a conflict may be relatively low (“Anne Johnson was afraid getting a mammogram would hurt”) or very high (“Anne Johnson was afraid she would die of breast cancer”).

Health-related narratives allow readers to see how a person like them has resolved a health-related conflict. In the story in the Appendix, the narrator describes a conflict between enjoying her family’s traditional recipes and wanting to eat more healthfully. She resolves the conflict by learning to prepare healthier versions of traditional dishes. Portraying resolution of the conflict as a process of incremental steps rather than an immediate change allows readers to see how behavior change might unfold in their own lives.

**Shape the story**. In *The Story Template,* Deardon writes, “Without good structure, the story tends to meander without a point” (30). Knowing the central conflict is a helpful starting point, but people constructing stories must still decide how to best present a series of events. Stories can start in the middle of the action, for example, with a dramatic event such as a diagnosis. This technique can be an effective way to draw readers in; background information and past events can be shared after readers are emotionally invested in the story. Stories can also be presented chronologically, with events recounted in order (29). This form may be especially effective if the purpose of a story is to describe a series of actions in sequence, such as a treatment protocol that takes place in a particular order.

If more than 1 narrative is used, writers must decide how to weave stories together. In some cases, it is effective to include narratives sequentially (eg, include 3 complete stories from breast cancer survivors). This technique is effective if 1 purpose of the stories is to have readers identify with people who had similar experiences. In other cases, it is effective to alternate perspectives (eg, give each woman’s perspective on diagnosis, followed by each woman’s perspective on treatment, followed by each woman’s perspective on survivorship). This technique is especially effective if the primary purpose is to focus on stages of a health condition or to compare a range of experiences, such as patient and caregiver perspectives on the same stage of the disease process. If uncertain about the best structure, story creators can try different structural choices during revision to see which the target audience finds most effective.

**Incorporate details**. In the writing manual *Writing Down the Bones*, Goldberg says simply, “Our details are important” (31). Informational texts usually focus only on the health topic at hand, despite evidence that contextual details in narratives can provoke strong emotional reactions (32). Vivid imagery is one method for “transporting” readers into a story (15). *Show, don’t tell* is a mantra from creative writing that also applies to health narratives. Showing details in a story allows a reader to feel and experience what the character feels and experiences. (28). In a story, a narrator may *tell* by simply summarizing: “It was hard sharing my cancer diagnosis with my son.” This flat statement is far less engaging than *showing *details of the scene: “When I told my 8-year-old I had cancer, he didn’t say anything for a minute. I could see there were tears in his big brown eyes, but he brushed them away with the back of his hand. Then he threw his arms around me and squeezed me hard.”

**Evoke emotion.** In *Bird by Bird: Some Instructions on Writing and Life,* Lamott advises, “[Y]ou must risk placing real emotion at the center of your work” (33). Emotional content is rarely included in academic or technical writing, but one advantage of narrative over informational text is the opportunity to elicit emotional responses from readers. Doing so enhances the recall of information and the vividness of the memory (34). Using appropriate details conveys emotion and allows readers to empathize with characters. As the authors of *Self-Editing for Fiction Writers* explain, when it comes to conveying emotion, “You don’t want to give your readers information. You want to give them experiences” (28). It is important to use emotion carefully in health-related narratives, however. Emotion should be related to the key health messages in a story; otherwise, it may distract readers from the messages (34).

**Use a natural voice.** Stories can be a bridge between readers and difficult medical concepts. It is important to use a voice that sounds natural to the target audience, which means using lay language and a conversational tone. Once a story is complete, read it aloud (1,28) to see if it flows and sounds natural. Use active rather than passive voice when choosing verbs (for example, “We *cooked* a lot of healthy dishes” instead of “A lot of healthy dishes *were cooked*”). In his book *On Writing*, novelist Stephen King admonishes writers to avoid using passive voice: “Two pages of the passive voice — just about any business document ever written, in other words, not to mention reams of bad fiction — make me want to scream” (35). King encourages writers to use direct sentences with active, vigorous verbs. Mary Pipher laments the “blandness and caution endemic to [the writing of] many academics” and tells writers, “Be bold. Be honest” (1). In the story in the Appendix, for example, the narrator does not say, “An unhealthy diet may contribute to obesity and other health conditions.” Instead, she talks about her family and says bluntly, “The high amounts of fat, salt, and sugar they ate hurt them.” Such language is direct and effective.

**Solicit feedback and revise.** Supreme Court Justice Louis Brandeis said, “There is no great writing, only great rewriting” (36). Creative writers often share their work-in-progress with other writers and receive feedback about which story elements are working well and which need revision. It is important to share health narratives with colleagues who can offer professional feedback and with members of the intended audience. Pilot testing stories can assess realism, literacy demands, and ease of understanding, as well as the extent to which readers identify with characters and see the stories as credible sources of health information. If multiple stories are developed, pilot testing helps practitioners select which are most effective.

## Conclusion

Narratives are useful for health communication, but incorporating them into public health practice requires skills that may be new to public health professionals. The transdisciplinary approach of using techniques from creative writing helps practitioners use stories effectively to prevent chronic disease.

## Appendix

This story was developed by the Health Communication Research Laboratory at Washington University in St Louis for use in *Healthy Body, Healthy Soul,* a newsletter aimed at African American women.

My Family’s Traditions

Collard greens with ham hocks, mustard potato salad, candied sweet potatoes, and fried corn. Do those foods make you think about Sunday dinners? What about the neighborhood picnics you enjoy? Just the thought of the smell of a pot of greens on the stove or the sweet smell of spices from brown sugar-glazed sweet potatoes baking in the oven makes me hungry.

All of these foods are part of the African American tradition of using food to hold on to tradition, as a source of comfort and to bring families together. Fruits and vegetables are a traditional part of the African American culture. But many of the fruit and vegetables dishes I grew up eating were also high in fat, salt, and sugar.

As an adult, I saw family members struggle with their weight, and the heart disease, diabetes, high blood pressure, and cancer that can come with it. The high amounts of fat, salt, and sugar they ate hurt them. At first I quit eating my favorite dishes to avoid the fat. That meant I didn’t eat as many fruits and vegetables. Then I realized that I could just make a few changes, like using skinless turkey leg or wing instead of fatback to season my greens, using a little margarine instead of butter to flavor my vegetables, adding herbs and spices to foods instead of salt, and enjoying the natural sweetness of fresh fruit desserts.

*I know that my kids and grandkids will have sweet memories of traditional foods just like I did, but the foods they remember will be a lot healthier. Tradition is important, but nothing compares to my family’s health. Our* new *tradition will be our old foods with a healthier way of cooking them.*

Comments

This is a simple story designed to connect with a female audience about healthy food choices. The style and structure work together to tell a brief story about how the narrator came to change her eating habits.

**Writing Style:** The story is told from the first-person point of view of an unnamed narrator (in the newsletter, the story was accompanied by a photograph of an African American woman who was presumably the narrator). The overall style is informal; the narrator uses simple words such as “kids,” for example, and the first sentence is actually a sentence fragment. After the opening sentence, the narrator engages the reader with two rhetorical questions that build on supposed common ground between narrator and reader. Verbs are active (eg, “I quit,” “I realized,” “I saw,” “I know”). Sensory details, such as the smell of spices, draw the reader in and help both establish the conflict and resolve it (as when the narrator learns to enjoy “the natural sweetness of fresh fruit desserts”).

**Structure:** This story follows a simple structure: context, conflict, and resolution. In the first paragraph, the foods and the pleasant memories they evoke establish the context and create a connection with the audience. The conflict arises at the end of the second paragraph: Many of these favorite foods are unhealthy. The third paragraph develops the conflict by showing the impact of the unhealthy foods on the narrator’s family members. Finally, the narrator resolves the conflict in the third and fourth paragraphs by learning how to make healthier versions of traditional foods.

**Encouraging action:** In the newsletter, this story was accompanied by a recipe for mixed greens casserole. Readers who were drawn into the story and motivated to change their food behavior were given a simple next step they could try.
